# Greater tuberosity fractures of the humerus: complications and long-term outcomes after surgical treatment

**DOI:** 10.1007/s00590-024-03969-9

**Published:** 2024-04-30

**Authors:** Diogo Nóbrega Catelas, Lucinda Correia, Filipa Adan e Silva, Ana Ribau, Rui Claro, Luís Henrique Barros

**Affiliations:** 1 Department of Orthopedic Surgery, Centro Hospitalar Universitário de Santo António, 4099-001 Porto, Portugal; 2 Shoulder Unit, Department of Orthopedic Surgery, Centro Hospitalar Universitário de Santo António, Porto, Portugal; 3 School of Medicine and Biomedical Sciences – University of Porto (ICBAS-UP), Porto, Portugal

**Keywords:** Greater tuberosity fractures, Proximal humerus, Surgical treatment

## Abstract

**Background:**

Isolated greater tuberosity (GT) fractures typically occur in younger patients following high-energy trauma compared to humeral neck fractures. Surgical treatment is indicated when superior displacement is > 5 mm. This study aimed to assess the complications and long-term outcomes of surgically-treated GT fractures.

**Methods:**

A retrospective review of 39 patients who underwent surgery from 2010 to 2014 was conducted. The cohort comprised 54.6% females, with an average age of 56.74 years and a median follow-up of 6.71 years. Only 25 patients returned for reevaluation, with functional outcomes assessed using Constant-Murley score.

**Results:**

Women were older than men (63.00 ± 12.15 vs. 48.65 ± 16.93, *p* = 0.006). 18/39 patients (46.1%) sustained avulsion-type, 1 patient out of 39 (2.6%) depression-type, and 20/39 patients (51.3%) split-type fractures. The mean Constant-Murley score was 84.08 ± 18.36, with higher scores observed in men (*p* = 0.021). Avulsion-type fractures were related to higher postoperative scores compared to split fractures (*p* = 0.069). Post-surgical complications occurred in 20.5% of patients, with no differences noted between sexes, fracture types, or procedures.

**Conclusion:**

This study enhances understanding of the long-term outcomes of surgically-treated GT fractures, aiding in treatment selection. Interfragmentary screws may be preferable in younger male patients, but are associated with the higher risk of reintervention, particularly in fragile bone. Prospective multicentric studies are warranted to further elucidate long-term results and treatment strategies.

## Introduction

Proximal humerus fractures are the third most common osteoporotic fractures, after proximal femur and wrist fractures [1, 2], constituting 5% of all fractures [3]. Typically occurring in older patients following low-energy trauma, these fractures affect women three times more frequently than men [4].

Conversely, greater tuberosity (GT) fractures typically occur in younger patients compared to the surgical humeral neck factures, often following high-energy trauma, with a higher incidence among men [4, 5]. Despite this, the majority of GT fractures (up to 95%) are minimally displaced (< 5 mm of superior displacement) and can me managed non-surgically [4, 6]. Isolated GT fractures account for 20% of proximal humeral fractures [1, 3, 7, 8]. It is noteworthy that the GT constitutes a critical anatomic structure of the shoulder for abduction and external rotation [3, 7].

**Fig. 1:**
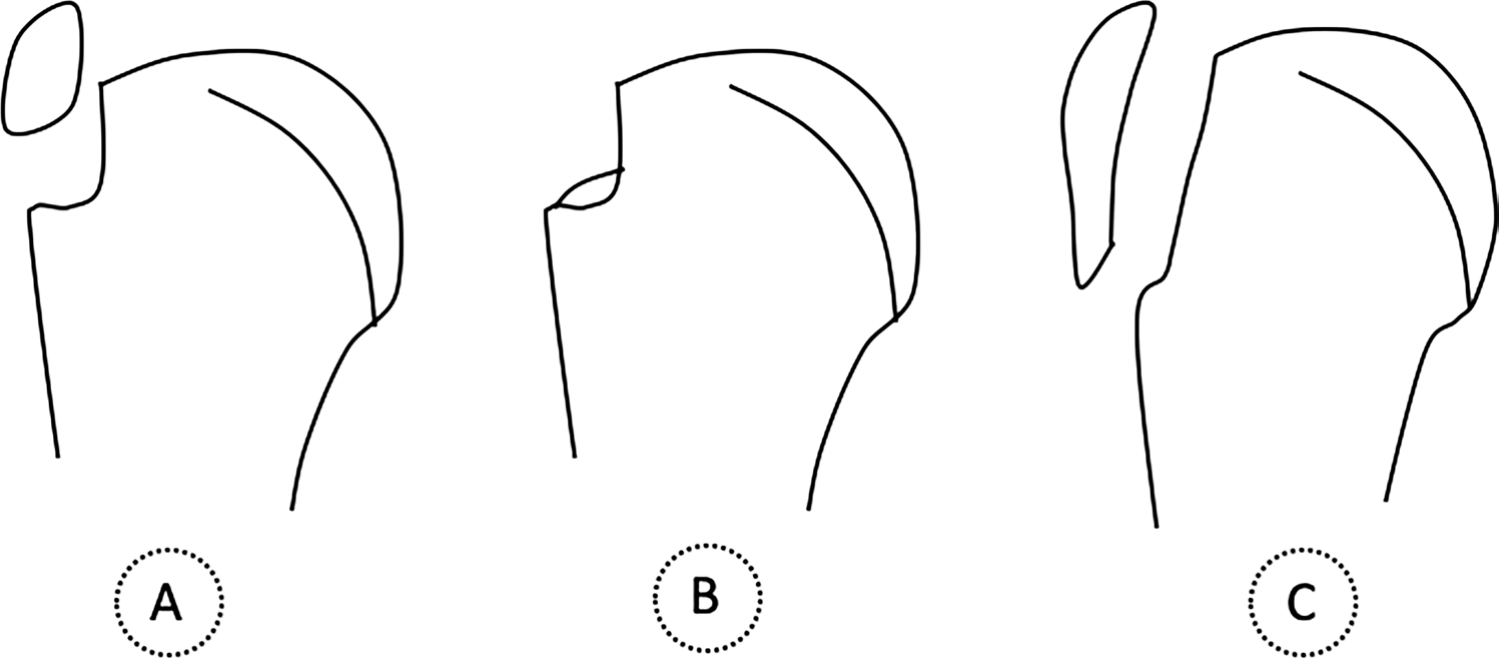
Illustration of Mutch morphological classification of the greater tuberosity fractures in the proximal humerus. **A** Avulsion-type; **B** Depression-type; **C** Split-type

**Fig. 2:**
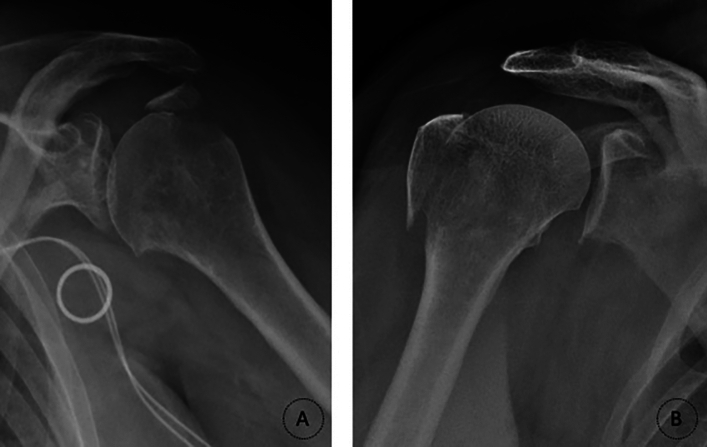
Radiographs illustrating Mutch classification of the greater tuberosity fractures in the proximal humerus. **A** Avulsion-type; **B** Split-type

Several classification systems have been devised to guide treatment. Neer and the AO foundation were the most popular [3, 8]. These two classifications address only one type of GT fracture, namely those with a large fragment with a vertical fracture line [3, 8]. Hence, in 2014, a morphological classification with implications on treatment was proposed by Mutch et al. (Figs. 1 and 2) [3, 8], which categorized GT fractures into avulsion fractures (involving small bone fragments and a horizontal fracture line, accounting for 39% of GT fractures), depression fractures (characterized by inferiorly displaced fragments, representing 20%), and split fractures (corresponding to those included in Neer and AO foundation systems, constituting 41%) (Figs. 1 and 2).

Surgical treatment is indicated when superior GT displacement is > 5 mm (observed in up to 15% of cases), as it results in better functional outcomes in such instances [4, 6]. However, in young active patients with prolonged overhead activity, even 3 mm of superior displacement might warrant surgical consideration [4].

Posterior displacement of GT fractures also significantly affects functional outcomes greatly, irrespective of superior displacements [4, 9]. Nevertheless, the threshold of posterior displacement necessitating surgical intervention remains undetermined [4].

**Fig. 3:**
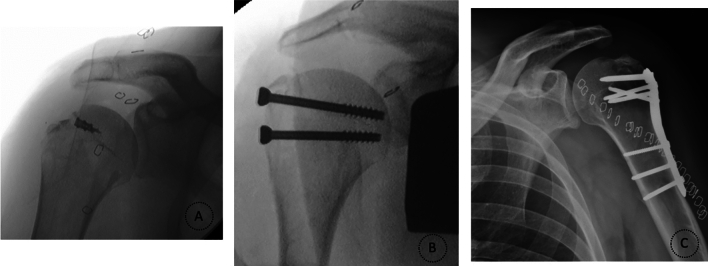
Radiographs showing surgical management of the greater tuberosity fractures in the proximal humerus. **A** Anchor repair; **B** Interfragmentary screws; **C** Locking plate fixation

Rouleau et al. [4] advocate a transosseous repair or a double-row anchor repair for avulsion-type fractures (Fig. 3). Depression fractures are usually managed non-surgically [4, 8]. Split fractures can be addressed with small locking plate fixation, double-row anchor repair, or interfragmentary screw fixation [4, 8] (Fig. 3).

In this work, the authors aimed to report how surgically indicated GT fractures are treated at our institution, what complications were faced following surgery, and the functional outcomes subsequent to surgical intervention.

## Material and methods

### Data source

Following institutional board approval (Comissão de Ética do Centro Hospitalar Universitário de Santo António/Instituto de Ciências Biomédicas Abel Salazar; N/REF.ª 2023.028(021-DEFI/022-CE), the institutional database was reviewed by the authors to retrieve GT fractures treated surgically from 2010 to 2014. CT scans had been performed in all operated patients to exclude humeral neck fractures before surgery.

A letter was sent to all patients to return for clinical and radiographic reevaluation. Written informed consent for participation in the study was obtained at the time of reevaluation. When patients were no longer reachable, clinical and radiographic data were evaluated.

**Fig. 4:**
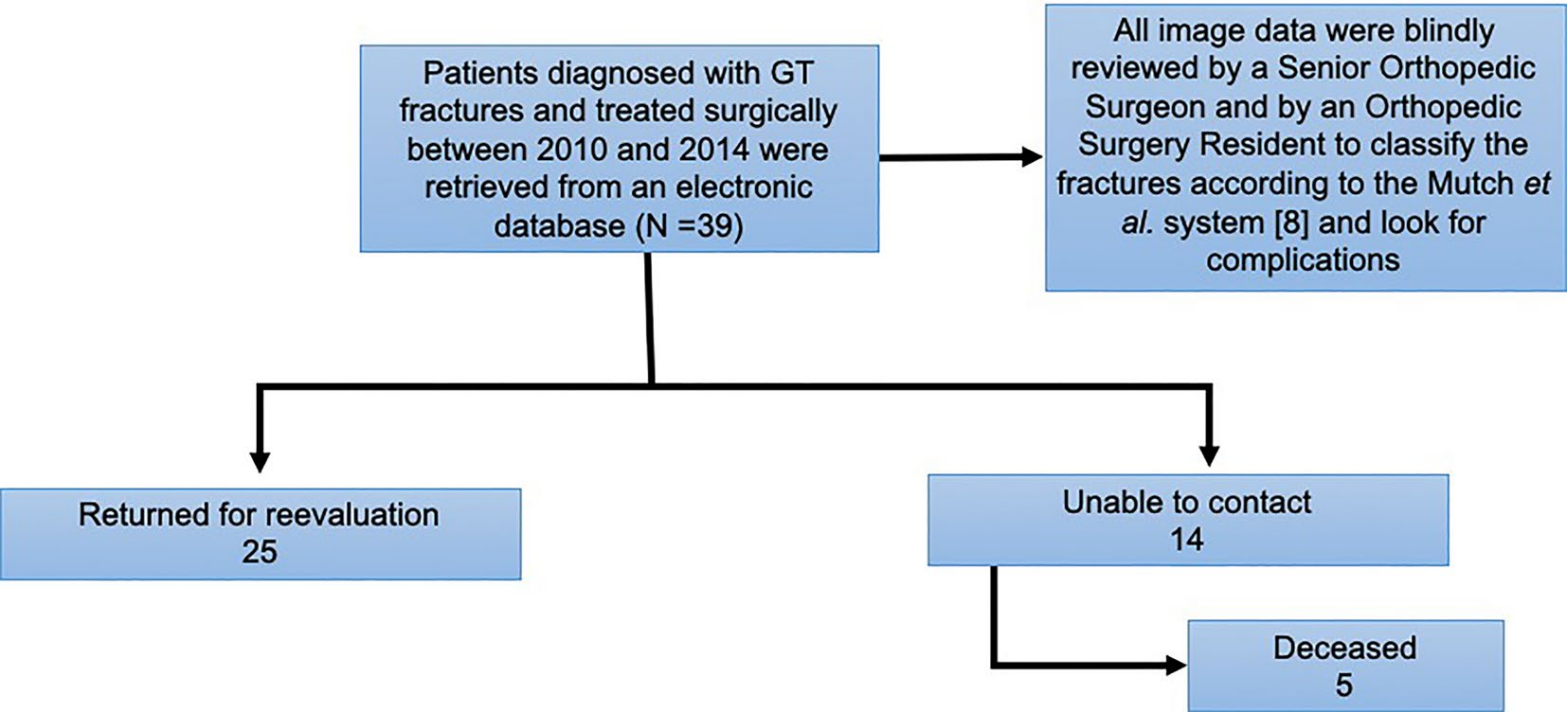
Review process of all GT fractures surgically treated

Figure 4 illustrates the review process of all patients diagnosed with GT fractures from 2010 to 2014 who were treated surgically at our institution. 39 patients were identified. 25 of these patients returned to the hospital to repeat the radiographs and for a functional evaluation. The authors were unable to reach 14 patients: 9 missed the appointment and did not answer the phone calls and 5 were deceased by the time this work was started. Radiographs were blindly reviewed by the Senior Orthopedic Surgeon (L.H.B.) and the first author (D.N.C), an Orthopedic Surgery Resident, to classify the GT fractures according to the Mutch system and to identify postoperative possible radiological complications. Cases of disagreement were reviewed and discussed among all authors. All 39 patients were included in the type of fracture, type of procedure and complications analysis, but only the 25 who returned for reevaluation were included in the functional analysis.

### Outcome measures

Patient charts were reviewed to obtain demographic data, including age, sex, comorbidities, follow-up duration, type of fracture, type of procedure, and complications. No patient had a previous history of ipsilateral shoulder surgery. At the time of reassessment, patients were clinically, functionally, and radiographically evaluated.

Functional outcomes were evaluated using a standardized and validated outcome score, the Constant-Murley score.

Standardized anteroposterior, lateral, and axillar shoulder radiographs were repeated at the time of reevaluation. Material failure or GT resorption were documented.

### Patient characteristics

The average patient age at the time of the injury and surgery was 56.74 ± 15.95 years (range 22–82 years; median 63 years) with a median clinical follow-up (including reassessment) of 6.71 years (range 1.17–12.75 years). Only patients with a minimum of 12 months of follow-up were included in the follow-up analysis. 8 patients were excluded: 2 died soon after the surgery (less than 2 months) due to other comorbidities not related to postoperative complications, and 6 were lost to follow-up (never attended any appointment, or attended only the first appointment after surgery). 17 patients were male (43.6%) and 22 were female (56.4%). Table 1 summarizes the baseline characteristics of the patients.

**Table 1:** Baseline characteristics of patients

	Male (*n* = 17; 43.6%)	Female (*n* = 22; 56.4%)	Entire cohort (*n* = 39)
*Age, years*			
Mean ± SD	48.65 ± 16.93	63.00 ± 12.15	56.74 ± 15.95
Range	22–74	39–82	22–82
*Follow-up*			
Mean	4.67	6.80	5.85
Range	0.33–12.33	0.33–12.75	0.33–12.75

### Statistical analysis

Data analysis was performed using Microsoft Office Excel 2016™ and SPSS Statistics V25™. Comparisons between groups were performed using independent sample *t* test, unpaired two-tailed *t* test, Mann–Whitney *U* test, One-way ANOVA, Kruskal Wallis test, and Fischer’s exact test. A *p* value < 0.05 was considered significant.

## Results

### Patient recruitment

This study comprised 22 females (56.4%) and 17 males (43.6%). Women who underwent a surgical treatment for a GT fracture were, on average, older than men (63.00 ± 12.15 vs. 48.65 ± 16.93, *p* = 0.006) (Table 1).

As detailed, radiographs were assessed by a Senior Orthopedic Surgeon and a Resident, and the fractures were classified into avulsion, depression, or split, in accordance with the Mutch classification. Among the 39 GT fractures included in the study, 18 out of 39 patients (46.1%) presented avulsion factures, 1 patient out of 39 (2.6%) had a depression fracture, and 20 out of 39 patients (51.3%) had split fractures.

Split and avulsion fractures were, respectively, the most prevalent in women (59.1%) and men (58.8%), *p* = 0.267. Patients with avulsion fractures were, on average, 52.89 ± 16.64 years old, and patients with split fractures were, on average, 60.70 ± 15.01 years old (*p* = 0.137).

Patients underwent one of four means of open fixation, through a deltopectoral approach: transosseous repair (*n* = 1, 2.6%), double-row anchor repair (*n* = 15, 38.5%), interfragmentary screw (*n* = 16, 41%), or locking plate fixation (*n* = 6, 15.4%) (Fig. 3). In one case, the supraspinatus tendon was excessively retracted, rendering fixation of the GT impossible; in this case, only a biceps tenodesis was performed. This particular patient was referred to our hospital 3 months after the trauma, explaining the tendon retraction.

When considering the procedure by sex, anchor repair was the most common option in women (45.5% of the cases), while interfragmentary screw was the most frequent choice in men (41.2%), *p* = 0.154. On average, patients treated with anchors were 61.40 ± 16.20 years old, those treated with a plate were 54.33 ± 15.25 years old, and the ones treated with screws were 52.38 ± 16.41 years old (*p* = 0.562).

Regarding the procedure based on the type of fracture: in the avulsion group, 10 patients were treated with anchors, 6 with interfragmentary screws, and 1 underwent a transosseous repair; in the split group, 5 were treated with anchors, 9 with interfragmentary screws and 6 with a plate (*p* = 0.017). The sole patient with a depression fracture underwent fixation with an interfragmentary screw.

### Functional outcomes

Function was assessed according to the internationally validated Constant-Murley score, which comprises four modules: pain, activities of daily living, active range of motion (ROM), and strength. Therefore, utilizing a summative scale (0–100), this score offers measures of physical impairments in ROM and strength, as well as patient-reported pain and activity limitation.

**Fig. 5:**
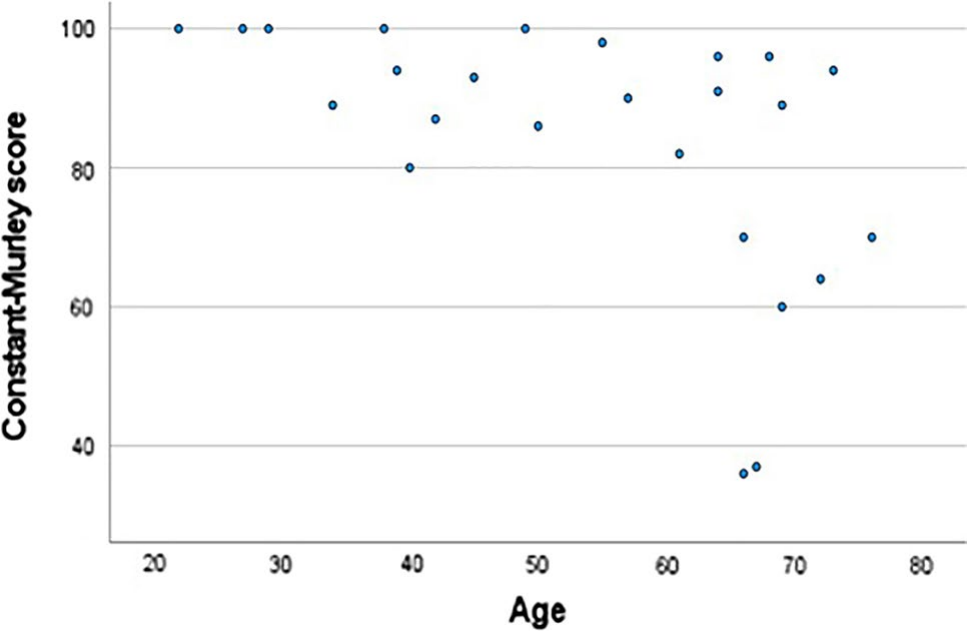
Constant-Murley score by age at the moment of surgery

25 patients were evaluated with this score (14 females and 11 males). The mean score was 84.08 ± 18.36, ranging from 36 to 100 (76.57 ± 21.17 for women and 93.64 ± 6.87 for men, *p* = 0.021). Among older patients, the score tended to be lower, as depicted in Fig. 5 (*p* = − 0.526).

In our cohort, patients with avulsion-type fractures exhibited higher postoperative scores (88.89 ± 17.11) compared to those with split fractures (81.38 ± 17.29), *p* = 0.069.

In this study, patients treated with anchors had the highest scores (91.14 ± 9.97), followed by those treated with screws (82.09 ± 21.56), *p* = 0.134.

### Complications and reoperations

**Fig. 6:**
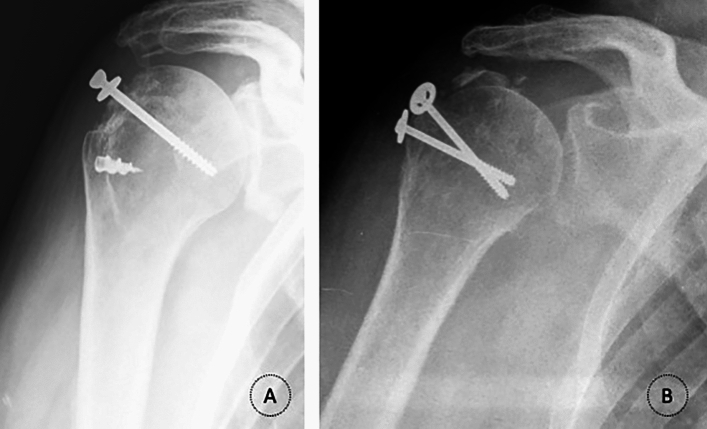
Radiographs displaying examples of complications. **A** Screw pull-out; **B** interfragmentary screw failure with greater tuberosity ascension

Post-surgical complications occurred in 8 patients (20.5%), with half of them (*n* = 4, 10.26%) necessitating revision (Fig. 6). Among these cases, one patient experienced anchor failure, but declined further surgery. Another patient exhibited GT resorption (with preserved function), one presented axillary nerve neuropraxia, and one had a slight reduction loss, none of which required reintervention. The 4 reinterventions comprised: extraction of an interfragmentary screw following failure (pull-out without fragment deviation; Fig. 6), extraction of a plate due to impingement (plate position was too high), revision with a plate after interfragmentary screw failure (Fig. 6), and inverted total shoulder arthroplasty following pseudoparalytic shoulder, GT resorption and supraspinatus rupture after initial treatment with an interfragmentary screw.

Postsurgical complications occurred in 5 women and 3 men (*p* = 0.704). There were no significant differences in terms of age (*p* = 0.522).

There were postsurgical complications in 3 patients with avulsion fractures and 5 with split fractures (*p* = 0.261).

Regarding the procedure, 3 patients treated with anchors experienced postsurgical complications, as did 4 treated with screws and 1 with a plate (*p* = 0.871).

Concerning scores and complications, patients without complications had higher scores (85.5 ± 16.40) compared to those with lower scores (78.4 ± 26.36), *p* = 0.785.

## Discussion

The authores conducted a study to assess the outcomes and complications of surgical treatment for GT fractures of the humerus by reviewing the institutional database from 2010 to 2014 and evaluating 39 patients who underwent surgical treatment. Among them, 25 patients returned for a clinical and radiographic reevaluation. The fractures were classified according to the Mutch system (Figs. 1 and 2) and the Constant-Murley score was used to evaluate functional outcomes (Fig. 5).

Surgical treatment of GT fractures aims to provide mechanical stability to enable early postoperative mobilization, by restoring anatomy and ensuring the rotator cuff tendon integrity [3]. Various surgical procedures have been described, so it’s important to choose the right option in accordance with fracture morphology, displacement, and comminution, and with patient’s characteristics [4].

Proximal humerus fractures, where GT fractures are included, are more frequent in women [4], and our study validated that finding of previous studies (53.4% vs. 43.6%, Table 1), even though women did not represent three times the men. Therefore, our study revealed a different gender distribution, possibly due to the inclusion criteria focusing solely on surgically treated GT fractures, which are more prevalent in younger patients with high-energy trauma, where men predominate, thus raising the proportion of men in our cohort.

As anticipated, women were, on average, older than men, and the results were statistically significant (*p* = 0.006). In fact, proximal humerus fractures typically affect older women with osteoporotic bone following low-energy trauma [1, 2], and younger men following high-energy trauma [4].

As aforementioned, Mutch et al. [8] proposed the following classification for GT fractures (with the following incidences): avulsion (39%), split (41%) and depression (20%) (Figs. 1 and 2). Our study corroborated these finding, with split fractures being the most frequent (51.3%), followed by avulsion fractures (46.1%). The lower proportion of depression-type fractures (1 case, 2.6%) in our study is attributed to the rarity of surgical intervention for this fracture type [4].

When comparing treatment options between sexes, anchor repair was the most frequent choice for women and interfragmentary screws for men (45.5% and 41.2%, respectively, *p* = 0.154) (Fig. 3). The screw fixation is cheap and remains cost-effective when indicated, but there is the concern of suboptimal biomechanical strength in fragile bone [3, 4]. So, this trend may be explained by the stronger bone quality typically seen in younger male patients, making them suitable candidates for screw fixation. In fact, corroborating this statement, patients treated with anchors were, indeed, on average, older than the ones treated with screws (61.40 ± 16.20 years vs. 54.33 ± 15.25, *p* = 0.562). The lack of statistical difference in age and treatment type analysis may be attributed to the small cohort size.

Avulsion fractures were predominantly treated with anchor repair (59%), whereas split factures were commonly managed with interfragmentary screws (45%), and the results were statistically different (*p* = 0.017), reflecting the suitability of each fixation method based on fracture characteristics. In fact, avulsion fractures generally result in a small fragment not amenable for screw fixation. Our data clearly follows a trend: younger patients, male patients, with better bone quality, and bigger fragments (split-type fractures, usually) tend to be successfully treated with interfragmentary screws that achieve optimal biomechanical strength and results in these patients; older patients, female patients and smaller fragments are equally successfully treated, but in these cases with anchor repair as the main form of fixation.

Functional outcomes assessed by the Constant-Murley score demonstrated statistically higher scores in men compared to women (93.64 ± 6.87 and 76.57 ± 21.17, respectively; *p* = 0.021). Various factors may contribute to this difference, including age-related capabilities and the influence of general health on functional outcomes: on the one hand, men were younger and therefore more capable of pursuing a better rehabilitation process; on the other hand, three of the four modules of the score (activities of daily living, active range of motion, and strength) may be affected by general condition and not only postsurgical incapability and, in this sense, as women were older as a group, we believe they would have obtained poorer scores regardless. As shown in Fig. 3, older patients tend to have lower scores, indeed. Pattern standard deviation was also much bigger in women denoting a bigger variability in function among women.

Patients with avulsion fractures achieved higher scores than the ones with split fractures, approaching statistical significance (88.89 ± 17.11 vs. 81.38 ± 17.29, *p* = 0.069). Avulsion fragments are usually smaller and possibly part of the tendon remains inserted to the non-fracture part of the GT, which might explain these findings.

Furthermore, patients treated with anchors had higher scores, on average, compared to those treated with screws (91.14 ± 9.97 and 82.09 ± 21.56, respectively, *p* = 0.134), although statistical significance was not reached, This finding may be confounded by the preference for anchor repair in avulsion fractures, which tend to have better functional outcomes.

No significant differences in complications were observed between sexes, fracture types, or treatment methods (*p* = 0.522, *p* = 0.261, and *p* = 0.871, respectively). Patients without complications tended to achieve higher scores, although statistical significance was not reached (85.5 ± 16.40 vs. 78.4 ± 26.36, *p* = 0.785). Notably, 3 of the 4 reinterventions occurred following interfragmentary screw failure, suggesting potential limitations of this fixation method.

As mentioned, at reevaluation, the 25 patients that returned repeated the radiography (AP and axillary views), revealing no additional complications beyond those identified during the original follow-up.

This study contributes to the limited literature on long-term outcomes following surgically-treated GT fractures. In fact, we found only one study with a bigger cohort, and none with a bigger mean follow-up, even though we included patients with follow-ups of only 12 months, unlike other studies we found (6.71 years; range 1.17–12.75 years).

Despite these facts, we recognize several limitations. Most GT fractures are minimally displaced and can be treated non-surgically, and, therefore, our cohort was still small. Loss to follow-up was significant and limited the number of patient functional outcome assessments that we were able to obtain. We can’t exclude the possibility of some of these patients seeking further treatment in other institutions, which would result in a higher number of reinterventions than we estimated.

## Conclusion

This study sheds light on the outcomes and complications of surgical management for GT fractures of the humerus. Our findings underscore the importance of tailored treatment approaches based on fracture characteristics and patient demographics.

Both interfragmentary screws and anchor repair appear to be viable options for GT fixation, but careful selection is paramount. Interfragmentary screws appear to be more suitable for male and younger patients with better bone stock and split-type fractures, while anchor repair may be preferable for female and older patients, with avulsion-type fractures and/or poorer bone stock.

This work further contributes to the understanding of the long-term outcomes of surgically-treated GT fractures, also considering the type of procedure selected. Given the relatively low incidence of GT fractures requiring surgical treatment, we believe a prospective multicentric study would help further comprehend long-term results in these patients and determine the optimal surgical approach for each specific case.

## References

[1] Shaw L et al (2019) The incidence of occult and missed surgical neck fractures in patients with isolated greater tuberosity fracture of the proximal humerus. BMC Musculoskelet Disord 20(1):482 31656189 10.1186/s12891-019-2810-y PMC6815442

[2] Bell JE et al (2011) Trends and variation in incidence, surgical treatment, and repeat surgery of proximal humeral fractures in the elderly. J Bone Joint Surg Am 93(2):121–131 21248210 10.2106/JBJS.I.01505 PMC3016042

[3] Chang CJ et al (2021) Augmented cerclage wire improves the fixation strength of a two-screw construct for humerus split type greater tuberosity fracture: a biomechanical study. BMC Musculoskelet Disord 22(1):350 33845833 10.1186/s12891-021-04215-7 PMC8042700

[4] Rouleau DM, Mutch J, Laflamme GY (2016) Surgical treatment of displaced greater tuberosity fractures of the humerus. J Am Acad Orthop Surg 24(1):46–56 26700632 10.5435/JAAOS-D-14-00289

[5] Gruson KI, Ruchelsman DE, Tejwani NC (2008) Isolated tuberosity fractures of the proximal humeral: current concepts. Injury 39(3):284–298 18243203 10.1016/j.injury.2007.09.022

[6] Williams GR Jr, Wong KL (2000) Two-part and three-part fractures: open reduction and internal fixation versus closed reduction and percutaneous pinning. Orthop Clin N Am 31(1):1–21 10.1016/S0030-5898(05)70124-3 10629329

[7] Court-Brown CM, Garg A, McQueen MM (2001) The epidemiology of proximal humeral fractures. Acta Orthop Scand 72(4):365–371 11580125 10.1080/000164701753542023

[8] Mutch J et al (2014) A new morphological classification for greater tuberosity fractures of the proximal humerus: validation and clinical implications. Bone Joint J 96-B(5):646–651 24788500 10.1302/0301-620X.96B5.32362

[9] Verdano MA et al (2014) Isolated fractures of the greater tuberosity in proximal humerus: Does the direction of displacement influence functional outcome? An analysis of displacement in greater tuberosity fractures. Acta Biomed 84(3):219–228 24458167

